# COVID-19 vaccine hesitancy: A Systematic review of cognitive determinants

**DOI:** 10.34172/hpp.2023.03

**Published:** 2023-04-30

**Authors:** Sara Pourrazavi, Zahra Fathifar, Manoj Sharma, Hamid Allahverdipour

**Affiliations:** ^1^Research Center of Psychiatry and Behavioral Sciences, Tabriz University of Medical Sciences, Tabriz, Iran; ^2^Health Education & Promotion Department, Tabriz University of Medical Sciences, Tabriz, Iran; ^3^Department of Library, Tabriz University of Medical Sciences, Tabriz, Iran; ^4^Department of Social and Behavioral Health, University of Nevada, Las Vegas (UNLV), Las Vegas, NV 89119, USA; ^5^Department of Internal Medicine, Kirk Kerkorian School of Medicine at UNLV, Las Vegas, NV 89154, USA

**Keywords:** COVID-19 vaccines, Vaccination hesitancy, Cognitive psychology, Systematic review

## Abstract

**Background:** Although mass vaccination is considered one of the most effective public health strategies during the pandemic, in the COVID-19 era, many people considered vaccines unnecessary and, or doubted the effectiveness of the COVID-19 vaccine. This review aimed to tabulate cognitive causes of COVID-19 vaccination hesitancy, which may help public health policymakers overcome the barriers to mass vaccinations in future pandemics.

**Methods:** For this systematic review, studies pertaining to COVID-19 vaccine hesitancy published up to June 2022 were retrieved from six online databases (Cochrane Library, Google Scholar Medline through PubMed, Scopus, and Web of Science). Inclusion criteria were the studies conducted on people who had a delay in accepting or refusing COVID-19 vaccines, reported the impact of cognitive determinants on vaccine hesitancy, and were written in English in the timeframe of 2020–2022.

**Results:** This systematic review initially reviewed 1171 records. From these 91 articles met the inclusion criteria. The vaccination hesitation rate was 29.72% on average. This systematic review identified several cognitive determinants influencing vaccination hesitancy. Lack of confidence and complacency were the most frequent factors that predicted vaccine hesitancy.

**Conclusion:** The identified prevailing cognitive determinants for COVID-19 vaccine hesitancy indicated that using initiative and effective communication strategies would be a determinant factor in building people’s trust in vaccines during the pandemic and mass vaccinations.

## Introduction

 The outbreak of the COVID-19 disease caused an emergency situation worldwide by affecting various aspects of human life. Although preventive measures, such as social distancing, wearing face masks in public, being under lockdowns, and quarantines helped to control COVID-19 virus transmission, returning to normal life urgently needed long-term solutions such as universal vaccination.^1^ COVID-19 vaccine reduced the mortality rate of disease and consequently had many benefits on the health and socio-economic aspects of life in the COVID-19 era.^2^ Additionally, the vaccines against the coronavirus changed the course of the pandemic to a better status by reducing the severity of COVID-19 disease and the incidence of new cases, even among unvaccinated people, through herd immunity.^2^ However, the COVID-19 vaccine, like all other new vaccines, faces the age-old public acceptance problem.^3^ Therefore, not only discovering and making available the COVID-19 vaccine is one of the critical challenges for the policymakers, but it will also be essential to encourage people to get it.^4^

 Even though the effectiveness and safety of many vaccines, such as COVID-19, have been well established, many people consider vaccines unnecessary and doubt their effectiveness and safety.^2^ Vaccine hesitancy is defined as a postponement in acceptance or denial despite the availability of a vaccine.^5^ It has been declared one of the top 10 warnings to attaining health for all by the World Health Organization (WHO).^2^

 Vaccine hesitancy has existed since the advent of the vaccines for influenza, human papillomavirus, polio, measles, etc.^3^ Recently, the world has witnessed people’s hesitation to receive the COVID-19 vaccine.^6^ COVID-19 vaccine hesitancy threatened doubtful people and the entire society by delaying the threshold of vaccine uptake necessary to achieve herd immunity.^2^ The acceptance rate of the COVID-19 vaccine in different countries varied from the lowest of 23.6% in Kuwait to 97% in Ecuador.^6^ In contrast, for successful control of COVID-19, the vaccine hesitancy should not be more than 25%-30%.^7^

 Many reasons can cause doubts about the COVID-19 vaccination, including fear of probable side effects, concern about the rapid vaccine production process, fear of inefficiency, unpleasant effect on some specific diseases,^5^ lack of trust in clinical trials, the sufficiency of the immune system to fight against COVID-19,^2^ the spread of fake information and news,^7^ religious beliefs,^8^ and political ideology.^9^ Therefore, the hesitancy of COVID-19 vaccination is not an individual problem; rather, it is a complex, multifaceted behavior that can have different cognitive, behavioral, social, and even political reasons in different societies and times. Although recent literature has investigated its reasons from different perspectives, little cumulative evidence has attempted to summarize in-depth and systematically the cognitive causes of COVID-19 Vaccine hesitancy. Therefore, the purpose of this study was to review the cognitive determinants of hesitancy toward COVID-19 vaccine.

## Materials and Methods

###  Study design and search strategy

 Six online databases (viz., Cochrane Library, Google Scholar Medline through PubMed, Scopus, and Web of Science) were searched thoroughly using a methodical approach in accordance with the Preferred Reporting Items for Systematic Review and Meta-Analysis (PRISMA) guidelines to identify relevant studies.^10^ We utilized the study’s research question to drive the search terms, namely, “what cognitive determinants influence COVID-19 vaccine hesitancy?” Therefore, the selected keywords were structured with Boolean operators. An example of this search strategy applied to the PubMed database is available in Supplementary file 1. After removing duplicates, the screening phase generated 723 articles. Moreover, we examined the references of identified publications for relevant studies.

###  Study selection

 Eligibility criteria were established beforehand using the PICO (population, intervention, comparison, and outcomes) design, and the research team (SP, ZF, HA) examined and approved the content validity:

*Populations*. Articles that included people who had delayed acceptance or refusal of COVID-19 vaccines despite its availability. No additional restrictions on population are considered.

*Comparison*. No criteria for comparison were applicable.

*Outcomes*. Any reported impact of cognitive determinants on vaccine hesitancy.

*Time*. All peer-reviewed journal articles published between January 2020 and June 2022 were included.

*Setting*. No limitations on the type of settings were imposed.

 English language quantitative (cross-sectional studies, randomized controlled trials, non-randomized studies, pre-post studies, and time series) or mixed methods (focused on the quantitative strand) research were eligible study designs. Systematic reviews were excluded but were employed to identify additional eligible studies.

 The search strategy was conducted in accordance with the Peer Review of Electronic Search Strategies statement.^11^ To ensure whether studies met the inclusion criteria, two authors conducted separate searches, screen the titles and abstracts, and then assessing the remaining 106 publications’ full texts.

###  Screening the full-text and synthesis

 For evaluation studies, information extracted included details about study characteristics, participants, setting, the prevalence of hesitation, and the findings related to the outcomes of interest.

 Two research team members, SP and ZF, independently pilot-tested the data extraction form utilizing two of the 106 articles and compared and discussed the findings. The feedback was used to refine the form. The final draft of the form was used by SP to extract data from the remaining 104 articles, which were independently checked by ZF. Title and abstract screening, along with full-text screening and cross-validation, were conducted by two review authors (SP and ZF) independently based on the abovementioned inclusion criteria. Any disagreements over a particular study were resolved through mutual discussion with a third reviewer (HA). Subsequently, 18 of the 106 articles were removed, resulting in a final included sample of 88 studies. Studies were excluded if they did not evaluate hesitancy toward COVID-19 vaccine and just measured vaccine acceptance. In addition, those studies which have not pointed out the role of cognitive determinants in hesitancy to the COVID-19 vaccine were eliminated.

**Figure 1:**
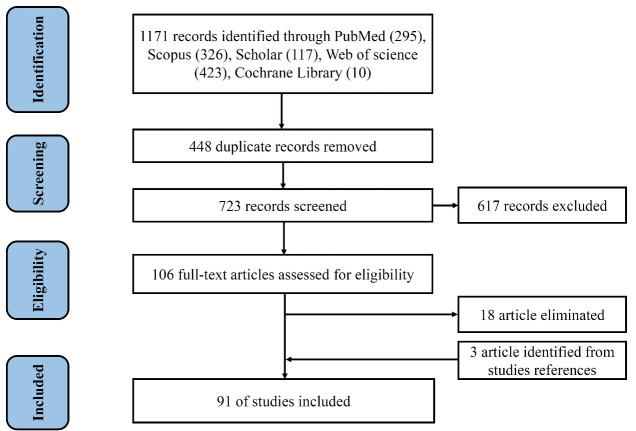
The diagram of the study based on PRISMA checklist

 We added three additional articles to our enumeration by reviewing the references from the articles. Figure 1 depicts the selection process over four-rounds. Using the PRISMA flow diagram, the documentation and summarization of the identification, screening, eligibility, and selection processes was done. Finally, at total of 91 articles were independently reviewed by SP and ZF. After that relevant data were extracted, and if there were any discrepancies, they were resolved for 100% agreement.

###  Quality assessment

 Strengthening the Reporting of Observational Studies in Epidemiology (STROBE) statement was used to conduct qualitative assessment independently with the help of two reviewers (SP and ZF).^12^ This checklist incorporates 22 criteria. If a study meets a condition, it receives one point, or zero if it is not or only partially disclosed. In this rating a higher overall score means that there is less of methodological bias. We divided each study’s risk of bias score by 22 (the highest possible score) and then multiplied it by 100 to assess the proportional percentage of fulfilled criteria. Any dissenting issues between the reviewers were resolved through discussion and consensus with the help of a third reviewer (HA). Studies’ quality were then sorted into excellent (matching ≥ 85% criteria), good (matching 70 to < 85% criteria), fair (matching 50 to < 70% criteria), and poor (matching < 50% criteria).^13^

## Results

###  Descriptive findings

 This review considered 91 peer-reviewed publications. The investigation comprised COVID-19 vaccine hesitancy studies from 36 different countries. Most surveys were conducted in the United States (n = 15), followed by China and Italy (n = 8 for each country), and Bangladesh (n = 7). Numerous studies were carried out in more than one country.^14-18^ The study carried out among US households^3^ had the largest sample size (n = 459 235), while one study carried out among homeless people in the US had the smallest sample size (n = 90).^19^ Out of these 91 studies, 27 were conducted with the general population, 27 with adults, 11 with health care workers, 10 with students, 7 with patients, 3 with parents/guardians, and 9 with other people such as pregnant women, homeless people, and refugees (Table 1).^3,5,6,8,9,14-99^

**Table 1:** Cognitive determinants of COVID-19 vaccine hesitancy

**Author(s)**	**Population & country**	**Hesitancy rate**	**Results**	**Author(s)**	**Population & country**	**Hesitancy rate**	**Results**
Abedin, 2021^21^	3646 adults from Bangladesh	8.5 % reluctant	Confidence in the country’s healthcare system	Al-Sanafi & Sallam 2021^22^	1019 HCWs from Kuwait	9.0%	The belief that the virus had a human-made origin
Adane et al, 2022^23^	404 HCWs from Ethiopia	36.0% refused	Anti-vaccine attitudes Poor knowledge and perception	Al-Mistarehi et al, 2021 ^24^	2208 individuals from Jordan	-	Lack of trust in the vaccine and their companies Lack of enough information Fear of side effects Concerns about safety and effectiveness Anti-vaccine attitudes
Adigwe, 2021^25^	1767 individuals from Nigeria	-	Concerns about side effects	Alrajeh, et al, 2021^26^	401 adults from KSA	-	Perceived susceptibility Perceived benefits Perceived barriers Concerns about effectiveness, safety, false vaccination, and side effects
Aemro et al, 2021^27^	440 HCWs from Ethiopia	45.9% hesitate	Unclear information provided by public health authorities Low perceived threat Concerns about side effects	Alzubaidi, 2021^28^	669 students from UAE	31.8% hesitant	Risks perception versus vaccine benefits Concerns about safety and effectiveness Attitudes about the disease and its consequences Knowledge and awareness about the vaccine Personal, family, and community experience with vaccination and feelings of solidarity Perception of the pharmaceutical industry Lack of confidence in government policies
Afzal et al, 2022^29^	3759 HCWs from the US	-	Concerns about rushed vaccine development Fear of side effects Lack of trust in the people advocating for the vaccines Anti-vaccine attitudes	An et al, 2021^30^	854 students from Vietnam	-	Concerns about side effects, safety, effectiveness, and rushed vaccine development Fear of needles Low perceived susceptibility Lack of confidence in government
Aguilar Ticona et al, 2021^31^	985 non-pregnant participants from Brazil	26.1% were hesitant and 7.9% unsure	Concerns about effectiveness and side effects	Ashok et al, 2021^32^	264 HCW from India	-	Concerns about rushed vaccine development Lack of enough information
Al-Ayyadhi et al, 2021^20^	6943 adults from Kuwait	74.3% hesitant	Concerns about safety and side effects Believing conspiracy theories	Badr et al, 2021^33^	1208 adults from the US	526 people were hesitant	Low perceived susceptibility Perceived the vaccination process as being more convenient
Baccolini et al, 2021^34^	5369 students from Italy	22% to 29% hesitancy ranged	Low perceived susceptibility and severity Concerns about safety and effectiveness Concern for the emergency	Chaudhary et al, 2021^35^	410 patients and their attendants from Pakistan	47.3% were hesitant	Lack of knowledge Understanding the way vaccines work Concerns about vaccine efficacy, safety, and comfort in the vaccine administration
Balan et al, 2021^36^	1581 students from Italy	8% undecided group	Rushed vaccine development Vaccine barriers outweigh benefits Belief in natural immunity Lack of trust in the vaccine Lack of trust in the local and medical authorities	Costantino et al, 2021^5^	346 patients from Italy	25.2% were hesitant	Fear of adverse events Concerns about rushed vaccine development Not afraid of COVID-19 Uncertain of vaccine efficacy
Blanchi et al, 2021^14^	417 patients from Europe, France, and Italy	18.9% were hesitant	Confidence in getting the vaccine easily Concerns about side effects and efficacy Lack of trust in scientists and the healthcare system	de Sousa Á et al, 2021^37^	6843 individuals from Portugal	21.1% were hesitant	Perceived high stress Afraid of future repercussions of the disease Vaccine Conspiracy Beliefs and misinformation
Bolatov et al, 2021^38^	888 students from Kazakhstan	70.7%-75.5%	Trust in the opinions of close relatives Concerns about side effects, safety, effectiveness, and quality Belief in natural immunity	Du et al, 2021^39^	3011 reproductive women from China	8.44% children and 3,011 reproductive women were hesitant	Low perceived susceptibility Lower perceived benefit High perceived barriers
Bou Hamdan et al, 2021^40^	800 students from Lebanon	10% were hesitant	Concerns about vaccine safety The vaccine in agreement with their personal views Agreement with conspiracies Level of knowledge about COVID-19 disease and vaccine Disagreement with that symptomatic cases are the only carriers of SARS-CoV-2	Ebrahimi et al, 2021^41^	4571 adults from Norwegian	10.46% were hesitant	Perceived risk of vaccination Belief in the superiority of natural immunity Lack of confidence in government Fear of infecting significant others
Butter et al, 2022^42^	1599 adults from the UK	17.7% uncertain, 8.1% refuse	Low perceived susceptibility	Ehde et al, 2021^43^	359 Adults from the US	20.3% were hesitant	Low perceived susceptibility Low trust in the Centers for Disease Control and Concerns about side effects, vaccine approval process, and potential impact of the vaccine given their health conditions
El-Sokkary et al, 2021^44^	308 HCWs from Egypt	41.9% were hesitant	Perception for the severity of COVID-19 COVID-19 vaccine safety Anti-vaccine attitudes	Ghaffari-Rafi et al, 2021^45^	359 adult patients from US	-	Concerns about vaccine safety Self-perception of a preexisting medical condition contraindicated with vaccination
Fares et al, 2021^46^	385 HCWs from Egypt	51% undecided 28% refused	Lake of enough clinical trials Fear of side effects of the vaccine	Gomes et al, 2022^47^	3232 individuals from Portugal	11% were hesitant	Feeling agitated, sad, or anxious Low or no confidence in the health services’ response Perceived measures implemented by the government as inadequate Low perceived susceptibility Concerns about safety and effectiveness
Fedele et al, 2021^48^	640 individuals from Italy	50% not sure	Concerns about side effects, safety, and effectiveness Opposition to vaccines Other non-specific reasons	Griva et al, 2021^49^	1623 adults from Singapore	9.9% were hesitant	Concerns about side effects, safety, and rushed vaccine development. Low perceived threat Lack of trust in the vaccine Low perceived benefits Lower moral and subjective norms
Freeman et al, 2021^50^	5114 adults from UK	16.6% unsure 11.7% hesitant	Beliefs about a COVID-19 vaccine Mistrust	Hwang et al, 2021^51^	13021 individuals from Korea	39.8% were reluctant or refused	Concerns about safety and side effects Complacency toward COVID-19 Awareness of the preventive guidelines Lack of confidence in government No fear of COVID-19
Genovese et al, 2022^52^	4116 individuals from Italy	17.5% were doubtful.	Lack of trust in the vaccine Low perceived susceptibility Fear of side effects	Hossain, et al, 2021^53^	1377 individuals from Bangladesh	35.25% unsure 18.99% denied	Concerns about side effects, safety, and efficacy Against the vaccination program Afraid of taking injections Belief in natural remedies
Gerretsen et al, 2021^15^	7678 adults from US and Canada	The mean (SD) hesitancy 2.3/6.0 (1.6)	Low perceived seriousness Low perceived threat Low perceived susceptibility Mistrust in vaccine benefit Preference for natural immunity Lack of confidence in government Risk propensity Mistrust in others The negative impact of COVID-19 on mental health	Hossain et al, 2021^6^	1497 adults from Bangladesh	41.1% were hesitant	Perceived susceptibility and severity Perceived benefits and barriers Anti-vaccine attitudes Subjective norm Perceived behavioral control Anticipated regret Lack of trust in the vaccine Complacent Calculative Collective responsibility
Jain et al, 2021^54^	1068 students from India	10.6% were hesitant	Concern about safety and efficacy Lack of awareness regarding their eligibility for vaccination Lack of trust in the government	Li et al, 2021^55^	2196 students from China	41.2% were hesitant	Perceived severity Concerns about side effects and effectiveness
Kanyike et al, 2021^56^	600 students from Uganda	30.7% were hesitant	Concerns about side effects Low perceived threat Belief in acquiring immunity against COVID-19	Liddell et al, 2021^57^	516 refugees living from Australia	28.1% were hesitant	Trust barriers Lower logistical barriers Attitudes relating to low control The Risk posed by COVID-19
Khairat et al, 2022^58^	3142 adults from the US	Mean (SD) 8 (2.83) hesitant	Lack of trust in the vaccine Concerns about side effects Lack of confidence in government	López-Cepero et al, 2021^8^	1911 adults from the US	More than 6.5% no intent 11% unsure	Lack of trust in the vaccine Unafraid of getting COVID-19 Not worried about getting COVID-19 Barriers to getting the vaccine Concerns about efficacy, safety, and novelty The rigor of vaccine testing Lack of confidence in government
Knight et al, 2021^59^	762 individuals from UK	22% were hesitant	Confidence Complacency Convenience	Luk et al, 2021^60^	1035 individual from China	29.2% undecided 25.5% no intention	Concerns about safety, side effects, and effectiveness Knowledge of SARS-CoV-2 transmission Perceived danger of COVID-19
Kucukkarapinar et al, 2021^61^	3888 adults from Turkey	43.9%-58.9% Increased rate of vaccine hesitancy/refusal	Conspiracy thinking Less knowledge of prevention Reduced risk perception Higher perception of media hype Trust in the Ministry of Health and medical professional organizations	Marijanovic et al, 2021^62^	364 patients from Bosnia and Herzegovina	37.6% Not sure	Doubt about the results of clinical trials of COVID-19 vaccines
Kuhn et al, 2021^19^	90 homelessness from the US	48% were hesitant	Fear of side effects Rejection of all vaccines Less trust in COVID-19 information from official sources, media, and friends Perceived threat	McCarthy et al, 2021^9^	779 patients from Australia	30.6% were hesitant	Vaccine conspiracy theory Having higher perceptions of anomie Lack of confidence in government Low perceived health threats
Lee & You 2022^63^	1016 individual from South Korea	53.3% were hesitant	Perceived susceptibility perceived benefits Perceived barriers Lack of confidence in government	Moujaess et al, 2021^64^	111 Patients from Lebanon	30.6% were hesitant	Desire to know more about the consequences of the vaccine in other patients with cancer
Muhajarine et al, 2021^65^	9252 adults from Canada	13 % were unsure, and 11% refused	Low perceived threat Low perceived severity Not concerned about spreading the virus	Orangi et al, 2021^66^	4136 individuals from Kenya	36.5% were hesitant	Low perceived threat Concerns about side effects and effectiveness
Murphy et al, 2021^18^	Ireland = 1041 and UK = 2025 individual	35% hesitancy for Ireland 31% hesitancy for England	Low trust in scientists, healthcare professionals, and the state Negative attitudes toward migrants Lower levels of altruism Higher levels of conspiratorial Lower levels of agreeableness Higher levels of internal locus of control Lower levels of the conscientiousness Higher levels of neuroticism Belief in chance Beliefs about the role of powerful others	Patwary et al, 2021^67^	543 adults from Bangladesh	15% were hesitant	Perceived barriers Subjective norms Low perceived threat Anti-vaccine attitudes Less self-efficacy Concerns about side effects and effectiveness Lack of enough information Belief in natural immunity
Navarre et al, 2021^68^	1964 HCWs from French	46.6% opposition to vaccination	Lack of trust in health authorities	Park et al, 2021^69^	902 individuals from South Korea	20.8 % were hesitant	Low perceived threat Concerns about safety Affective and Cognitive risk perception of COVID-19 Perceived the government’s performance as ineffective
Nazlı et al, 2021^70^	467 18-65 years old from Turkey	13.2% were hesitant	Belief in conspiracy theories low fear of COVID-19	Paschoalotto et al, 2021^71^	1623 individuals from Brazil	30% were hesitant	Concerns about side effects
Nery et al, 2022^72^	2521 individuals from Brazil	18.6% were hesitant	Low perceived threat	Pedersen et al, 2021^16^	423 individuals from 31 countries	4% were hesitant	Concerns about side effects, rushed vaccine development, and effectiveness Lack of enough information
Nguyen et al, 2021^73^	651 pregnant women from Vietnam	-	Concerns about safety and effectiveness	Peirolo et al, 2021^74^	776 HCWs from Switzerland	-	Low perceived threat Concerns about side effects
Okubo et al, 2021^75^	23142 individuals from Japan	11.3% were hesitant	Concerns about adverse reactions Doubts about the vaccine efficacy Low perceived susceptibility	Prickett et al, 2021^76^	1284 individuals from New Zealand	14.2% were unlikely and 15.1% unsure	Concerns about the side and future effects Thought their chances of becoming seriously ill if they caught COVID-19 were low Being protected by herd immunity
Rahman et al, 2021^77^	850 adults from Bangladesh	30.23% were hesitant	Afraid of side effects lack of enough information Lack of trust in the vaccine	Schernhammer et al, 2022^78^	1007 adults from Australia	41.1% were hesitant	Optimism
Reno et al, 2021^79^	1011 individuals from Italy	31.1% were hesitant	Perceived threat	Shekhar et al, 2021^80^	3479 HCWs from the US	56% were hesitant	Concerns about Safety, efficacy, and rushed vaccine development
Roberts et al, 2022^81^	1004 adults from the US	-	Anti-vax beliefs	Shen et al, 2021^82^	2361 individuals from China	-	Lack of trust in the vaccine Risks perception
Ruggiero et al, 2021^83^	427 parents from the US	21.93% were hesitant	Concerns about side effects and safety	Soares et al, 2021^84^	1943 individuals from Portugal	56% wait and 9% refuse.	Lack of trust in the vaccine and the health service response Worse perception of government measures Perception of the information provided as inconsistent and contradictory
Schaal et al, 2021^85^	2339 pregnant & breastfeeding from Germany	Pregnant: 28.9% unsure Breastfeeding: 28.1% unsure	Scientific data on the COVID-19 vaccination are too preliminary Lack of enough information Being anxious because of vaccine damage to the unborn or causing pregnancy Complications	Solak et al, 2022^86^	525 adults from Turkey	-	Need for cognitive closure
Sharma et al, 2021^87^	428 African Americans from US	48% were hesitant	Perceived Advantages Perceived Disadvantages Participatory Dialogue Behavior Confidence	Spinewine et al, 2021^88^	1132 HCWs from Belgium	37.1% were hesitant	Concerns about side effects, rushed vaccine development, and effectiveness Low perceived threat
Schwarzinger et al, 2021^89^	1942 adults from France	71.2% were hesitant	Vaccine efficacy Concerns about side effects Communication about the collective benefits of herd immunity	Stojanovic et al, 2021^17^	32028 individuals from Brazil, Canada, Colombia, France, Italy, Turkey, UK, US	27% were hesitant. France had highest level of hesitancy (47.3%) and Brazil the lowest (9.6%)	Fewer COVID-19 health concerns Higher personal ﬁnancial concerns
Theis et al, 2021^90^	816 Wright-Patterson Air Force Base (WPAFB) from the US	22.7%	Concerns about side effects and effectiveness Vaccines making them feel sick Vaccine infects them COVID-19 Being worried about misinformation/political agenda	West et al, 2021^91^	360 Temporary Foreign Workers from Bangladesh	25% were hesitant	Fear of side effects Low perceived threat Willingness to take the vaccine by more people first Lack of enough information
Ticona et al, 2021^92^	985 individuals from Brazil	26.1% were hesitant	Concerns about effectiveness and side effects	Wu et al, 2022^93^	306 adult from the US	33.99% were hesitant	Concerns about side effects, safety, ingredients, rushed vaccine development, and effectiveness Low perceived threat Concerns about vaccine causing MS relapse, making MS medication ineffective, and getting the COVID-19 infection Prior bad experiences with other vaccines
Tram et al, 2021^3^	459235 households from the US	10.2% “probably NOT” get a vaccine	Concern about side effects and safety Other people need it more than I Lack of trust in the vaccine Lack of confidence in government	Xu et al, 2021^94^	4748 parents from China	25.2% of women, 26.1% of their spouses, and 27.3% of their children	Psychological distress Concern about safety
Turhan et al, 2021^95^	620 individuals from Turkey	-	Lack of trust in healthcare system	Yanto et al, 2021^96^	190 adults from Indonesia	13.2% were hesitant	Agreeableness trait Neuroticism Lack of confidence in government, scientists, and HCWs
Wang & Zhang 2021^97^	382 parents from China	-	Psychological flexibility Self-efficacy Coping style	Zhang et al, 2021^98^	1015 individuals from China	82 Doubtful 39 Strongly Hesitancy	Conspiracy beliefs Medical mistrust Knowledge of vaccines Vaccine confidence and complacency
Wang et al, 2021^99^	7318 adults from China	67.6% were hesitant	Confidence Complacent Convenience	-	-	-	-

HCWs: health care workers.

###  Risk of bias 

 On average the studies met 68.5% (range = 51-86%) of the rating criteria. On the whole, the studies showed a moderate risk of bias, and more than half of them (n = 63; 69%) were of good quality (range = 70 to 85).

###  Variations in vaccine hesitancy and refusal

 Vaccination hesitation rate varied from 4% among patients with primary ciliary dyskinesia^16^ to 74.3% (mean = 29.72) in people over 18 years of age living in Kuwait^20^ and reported refusal rates were 8.6% to 75.5% (mean = 26.88). In addition to the hesitancy rate, some studies also measured uncertainty (mean = 23.25), undecided (mean = 29.4), and reluctance (mean = 24.15).

###  Cognitive Determinants of COVID-19 Vaccine Hesitancy 

 Among the evaluated peer-reviewed literature, based on a collective sample of 1 335 139 participants, several categories of cognitive determinants were extracted:

###  5C Psychological Antecedents

 A number of studies have used in a way five factors of confidence, complacency, constraints, calculations, and collective responsibility, which are known as 5C psychological antecedents.^3,5,6,8,9,14-16,20,21,24-36,38-44,46-63,65-77,79,80,82-84,88,89,91-93,94-98^ Confidence and complacency in the vaccine were two of the most frequent variables used by most studies. We categorized the concerns about probable vaccine side effects, vaccine effectiveness, the rapid procedure of vaccine manufacturing, and lack of trust in the efficiency of some brands under the perceived confidence of participants about the COVID-19 vaccine. In addition, perceived threats, including perceived susceptibility and severity, the risk posed by COVID-19, and risk propensity, were categorized as complacency.

###  Perceived self-efficacy and perceived behavioral control

 According to studies, individuals with higher general self-efficacy and specific self-efficacy of preventing COVID-19 displayed stronger intentions to get vaccinated.^67,97^ In addition, in relation to perceived behavioral control, Hossain et al found that the respondents who registered voluntarily for COVID-19 vaccination had been less vaccine-hesitant.^6^

###  Perceived locus of control 

 Murphy et al used the locus of control variable as a psychological indicator of COVID-19 vaccine acceptance/hesitancy/resistance.^18^ They measured internal and external locus of control among Irish and England participants. Their results indicated that in the Irish and UK, vaccine hesitant/resistant people felt more control over their lives, acted based on their preferences, and had higher levels of internal locus of control.

###  Inhibiting subjective norms 

 Social/peer influence was the variable that some studies applied as a predictor of COVID-19 vaccination hesitancy.^6,49,67^ The results of their studies have shown that vaccine hesitancy tended to decrease with the increase of perceived subjective norms.

###  Anti-vaccine beliefs 

 We found that conspiracy theories concerning the COVID-19 vaccine have a significant impact on decision to hesitate. For example, some related beliefs were as follows: (i) Vaccine protection against COVID-19 is temporary; (ii) COVID-19 vaccines modify DNA; iii) the vaccine can induce other disorders such as autism or autoimmune diseases; (iv) COVID-19’s vaccine has chips implanted to control people; (v) the vaccine’s efficacy and published studies are untrue^37^; (vi) The virus is manufactured by humans ; (vii) the virus’s spread is an deliberate attempt to reduce the global population’s growth; and viii) COVID-19 is a biological weapon produced by China to crush the West.^50^

###  Stress and anxiety

 Perceived stress has been used as a factor associated with COVID-19 vaccine hesitancy by de Sousa et al in Portuguese-speaking countries. They found a significant direct relationship between vaccine hesitancy and perceived stress.^37^ According to Xu et al, parents with psychological distress are more likely to hesitate to vaccinate for themselves, their spouses, and their children.^94^ Feeling agitated, sad, or anxious were other factors that were shown to be associated with vaccine hesitancy in a survey conducted by Gomes et al.^47^

###  Fears and concerns 

 Some studies reported fears such as fear of needles and injection,^30^ fear of infecting significant others,^41^ and higher personal ﬁnancial concerns/fear of the expensive vaccination costs, which make people hesitate to adopt the COVID-19 vaccination. Additionally, the Ghaffari-Rafi et al study showed that patients with an insight into a preexisting medical condition believed that COVID-19 vaccination might threaten their health because of existing disease.^45^

###  Optimism

 Optimism indicates the extent to which people hold positive expectancies for their future^100^ used by Schernhammer et al. They explored the correlation of optimism with hesitancy toward COVID-19 and reported that persons with medium to high optimism were less prone to vaccine-hesitancy.^78^

###  Personality traits

 Some personality traits such as personal anomie, altruism, conscientiousness, agreeableness, and neuroticism have been used by several studies^9,18,96^ as psychological indicators of vaccine hesitancy. These studies indicated that higher levels of neuroticism, perceptions of anomie, and lower levels of agreeableness, conscientiousness, and altruism might influence the increase in COVID-19 vaccine hesitancy.

## Discussion

 This systematic review aimed to investigate the cognitive determinants of COVID-19 vaccination hesitancy. We discuss several cognitive factors that may play a role in COVID-19 vaccine hesitancy.

 Confidence and complacency, two antecedents of the 5C psychological model, were among the most common cognitive factors studied to explain COVID-19 vaccine hesitancy. The confidence was relevant to trust in the government’s decisions, the effectiveness of the vaccines, and the COVID-19 Vaccine delivery system.^101^ Confidence in the COVID-19 vaccine and concerns about its safety have been reported in most studies.^5,14,20,29,31,38^ According to studies, concerns about the probable side effects of the vaccine, its ingredients, its effectiveness, and safety, as well as the rapid process of vaccine production and the vaccines approval process, reduce the trust of people in the COVID-19 vaccine. Although most of the side effects of COVID-19 vaccines have been confirmed scientifically, some are undocumented or have fewer shreds of evidence. This can lead to insufficient knowledge, the formation of improper beliefs, incorrect information, and mistrust in vaccines.^102^

 When a vaccine is quickly produced and distributed, information sources such as the Internet and other social media disseminate claims about its harms and ineffectiveness.^103,104^ Much of this information may exaggerate risks associated with the COVID-19 vaccines^105^ and could cause the formation of anti-vaccine conspiracy beliefs.^106,107^ Most of the information that is published by unreliable sources targets the safety of vaccines, worries people about short-term adverse reactions and possible long-term effects of the COVID-19 vaccine, and can ultimately lead to hesitation and refusal to vaccinate.^105^

 On the other hand, confidence in vaccines can result from people’s trust in the public health care system and in delivering safe and effective vaccines.^101^ In this regard, the WHO vaccine advisory group highlights the role of healthcare workers in building confidence in COVID-19 vaccines. Because healthcare providers can be effective in improving people’s insights and awareness about the benefits of vaccination and addressing people’s concerns about newly developed vaccines.^108^

 The role of distrust of the government and health care system is significant in causing vaccine hesitancy.^28,30,41,97^ Usually, people are worried about the side effects of vaccines imported to the country or manufactured there, which may lead to a lack of trust and fear about vaccines. ^7^ The lower the people’s trust in the government, the more risk perception of the threat. Therefore, governments should provide safe vaccines.^69^ In fact, trust in the government and health authorities is essential for vaccine acceptance, especially in cases such as COVID-19, where anxiety about the nature of the disease is significant.^101^

 When the nature of a disease is not completely clear, the chance of spreading conspiracy beliefs may increase, and it was recognized that in the COVID-19 pandemic, the growth of conspiracy beliefs and the reduction of people’s participation in vaccination have occurred.^9^ Conspiracy theories explain the negative emotions and uncertainty that traditionally increase during times of social crisis (such as war, environmental disaster, and terrorism). In this situation, uncertainty, powerlessness, and fear and anxiety increase.^9^ With the rapid prevalence of the COVID-19 pandemic, a wide range of conspiracy beliefs emerged and spread. For example, COVID-19 is a hoax, a biological weapon developed by the Chinese, and the COVID-19 vaccine microchips will be injected to control COVID-19,^9,109^ which indicates that the vaccine manufacturing companies underestimate the side effects of the vaccines.^9^ The development of such beliefs may cause mistrust and reduce the vaccination acceptance rate. Therefore, delivering information that focuses on the effectiveness and safety of the COVID-19 vaccine from reliable sources can be influential in reducing vaccination hesitancy.

 The second antecedent of 5c psychological is complacency. More complacency is defined as a lower perceived threat of disease and the belief that vaccination is unnecessary as a preventive measure. In other words, people with high complacency have more feelings of invulnerability and less preventive behavior than those with low complacency.^101^ According to the Health Belief Model (HBM), people are most likely to take a preventative behavior when they perceive the threat of disease. The HBM is one of the most widely used models to explain vaccination behavior.^6,110^ Studies have shown that worrying about getting infected with COVID-19 and believing in the seriousness of its consequences can persuade people to get the COVID-19 vaccine.^6,111^ Also, the newer fourth-generation models, such as the multi-theory model of health behavior change, have underscored the role of getting convinced of the advantages of behavior change over the disadvantages and building behavioral confidence.^87^

 One of the important factors in getting the vaccine is the perceived benefits of a vaccine. Such as the belief in its protective effect against COVID-19 and its subsequent side effects are among the influential factors in adherence to the COVID-19 vaccine.^111^ In contradiction of that, perceived physical and psychological barriers that can make the vaccine an unpleasant experience ^21,111^ and concerns about safety and its probable side effects, fear of needles, and its costs can increase vaccination hesitancy.^112,113^

 Locus of control and belief in chance were other cognitive factors recognized in this study. Health locus of control refers to the degree to which a person believes that he/she, as opposed to external forces, has control over his/her health. Locus of control is conceptualized as internal or external.^114^ The internal dimension is positively associated with engaging in health behaviors, and chance as the external dimension is positively related to non-adherence to health behaviors.^115^ People whose health locus of control is external may be doubtful about how to behave in a healthy manner,^116^ such as vaccination, and it is reported that the external locus of health control is related to a lower level of childhood vaccination through parental attitudes.

 Studies have used self-efficacy and perceived behavioral control as predictors of COVID-19 vaccine hesitancy.^6,67,97^ As self-efficacy reflects one’s belief in their ability to perform a particular behavior,^110^ like the COVID-19 vaccination, perceived behavioral control similar to self-efficacy also refers to the person’s belief that the considered behavior is under control. As a result, most psychosocial health behavior theories postulated that self-efficacy and perceived behavioral control had been introduced as major determinants of engaging in health behavior.^110^ Also, the role of behavioral confidence has been underscored in the newer fourth-generation models, such as the multi-theory model (MTM) of health behavior change.

## Limitations

 Due to resource constraints needed to translate and retranslate studies published in other languages, the investigation was limited to manuscripts published in English only. Hence the results are not representative of research published in other languages. Further, the search in this review was limited to the title, keywords, and abstract of each publication. Perhaps more in-depth search could have resulted in identification of more studies. A single statistical analysis of the data was not practical or feasible because of the sizable variability in the cognitive determinants of COVID-19 across studies. Therefore, a narrative analysis was accomplished, thereby limiting the external validity of the conclusions.

###  Implications for practice and future research

 Given that hesitancy and distrust of a new health product and service such as the COVID-19, vaccine will always exist, the development of strategies that can build trust in people to vaccinate and improve the government’s ability to manage and successfully implement mass vaccination calls for attention. According to studies, several factors can contribute to building trust^117^:

*Responsiveness*: Health authorities should show competence in responding to people’s health needs, fears, and concerns by establishing a transparent and coherent relationship about the vaccine quality. Qualitative research can help identify people’s needs, concerns, and fears about the COVID-19 vaccination.

*Openness:* The public must understand the importance of rapid vaccine production and distribution to achieve herd immunity during new epidemics. Also, more importantly, people should ensure that no quality or safety standards have been sacrificed for speed in the vaccine production process. Therefore, people should be informed about all phases of production, approval, evaluation, and distribution of vaccination through a proper communication strategy. Paying attention to myths, misconceptions, and false information about vaccination, monitoring the messages of widely used social media such as the Internet, spreading correct information through the creation and introduction of reliable information sources, and increasing health literacy and e-health literacy of people are other strategies for considering openness.

*Reliability, integrity, and fairness:* Holding campaigns to encourage people to take the vaccine with the presence of health authorities, pioneering them in receiving the vaccine, and providing information about all the benefits and harms of the vaccine, will increase confidence in the vaccination.

## Conclusion

 COVID-19 vaccine hesitancy as a significant challenge for public health has been reported in many countries. Our findings highlight the importance of understanding the cognitive factors contributing to COVID-19 vaccine hesitancy to develop effective health communication programs for persuading people toward COVID-19 vaccination and the most common reason for vaccine hesitancy was a lack of confidence and complacency. Multiple factors, including concerns about vaccine safety and side effects, perceived susceptibility and severity, the risk posed by COVID-19, and risk propensity, could influence delay or refusal to accept the vaccine. Information through trusted sources to reduce hesitancy about the COVID-19 vaccination.

## Competing Interests

 Hamid Alahverdipour is Editor-in-Chief of the Health Promotion Perspectives. Other authors declare no competing interests.

## Ethical Approval

 This research was performed based on Tabriz University of Medical Sciences ethics committee approval (Approval ID: IR.TBZMED.REC.1400.564).

## Funding

 This study was supported by Tabriz University of Medical Sciences, Tabriz, Iran. The funders had no role in study design, data collection and analysis, the decision to publish, or the preparation of the manuscript.

## Supplementary Files

 Supplementary file 1 contains search strategy applied to the PubMed database.
