# Supplementary material for: COVID-19 vaccine hesitancy: A Systematic review of cognitive determinants
Source: Health Promot Perspect. 2023 Apr 30;13(1):21–35. doi: 10.34172/hpp.2023.03 (PMC10257562; doi:10.34172/hpp.2023.03)
Supplement: Supplementary file 1 — contains search strategy applied to the PubMed database. [file hpp-13-21-s001.pdf]

Pourrazavi et al, **Health Promotion Perspectives**, 2023, 13(1), 21-S1
doi: 10.34172/hpp.2023.03
https://hpp.tbzmed.ac.ir

Supplementry File 1. Search Strategy for the PubMed Database

("COVID-19 Vaccines"[Mesh]) OR (COVID 19 vaccine*[Title/Abstract]) AND (percept*[Title/Abstract] OR cognitive [Title/Abstract] OR psychological[Title/Abstract] OR conceptual[Title/Abstract]) AND (determinant*[Title/Abstract] OR factor*[Title/Abstract]) AND (acceptance[Title/Abstract] OR hesitancy[Title/Abstract] OR Uncertainty[Title/Abstract] OR confidence[Title/Abstract] OR engagement[Title/Abstract] OR barrier[Title/Abstract] OR rejection[Title/Abstract] OR fear[Title/Abstract] OR doubt[Title/Abstract] OR distrust[Title/Abstract] OR dropout[Title/Abstract] OR exemption[Title/Abstract] OR trust[Title/Abstract] OR mistrust[Title/Abstract] OR refusal[Title/Abstract] OR rejection[Title/Abstract] OR controvers*[Title/Abstract] OR opposition[Title/Abstract] OR delay[Title/Abstract] OR dilemma*[Title/Abstract] OR uptake*[Title/Abstract] OR concern*[Title/Abstract] OR perception[Title/Abstract])",,,"("COVID-19 Vaccines"[MeSH Terms] OR "COVID 19 vaccine*"[Title/Abstract]) AND ("percept*"[Title/Abstract] OR "cognitive"[Title/Abstract] OR "psychological"[Title/Abstract] OR "conceptual"[Title/Abstract]) AND ("determinant*"[Title/Abstract] OR ""factor*"[Title/Abstract]) AND ("acceptance"[Title/Abstract] OR "hesitancy"[Title/Abstract] OR "Uncertainty"[Title/Abstract] OR "confidence"[Title/Abstract] OR "engagement"[Title/Abstract] OR "barrier"[Title/Abstract] OR "rejection"[Title/Abstract] OR "fear"[Title/Abstract] OR "doubt"[Title/Abstract] OR "distrust"[Title/Abstract] OR "dropout"[Title/Abstract] OR "exemption"[Title/Abstract] OR "trust"[Title/Abstract] OR "mistrust"[Title/Abstract] OR ""refusal""[Title/Abstract] OR "rejection"[Title/Abstract] OR ""controvers*"[Title/Abstract] OR ""opposition"[Title/Abstract]

OR "delay"[Title/Abstract] OR "dilemma*"[Title/Abstract] OR "uptake*"[Title/Abstract] OR "concern*"[Title/Abstract] OR "perception"[Title/Abstract])".
